# Emergence and Development of Health Risk Communication Networks Among Street-Level Health Bureaucrats During the COVID-19 Pandemic Crisis in Myanmar

**DOI:** 10.1007/s13753-022-00431-4

**Published:** 2022-08-09

**Authors:** Thein Myomin, Seunghoo Lim

**Affiliations:** Public Management and Policy Analysis Program, Graduate School of International Relations, International University of Japan, Minamiuonuma, Niigata 949-7277 Japan

**Keywords:** Client meaningfulness, COVID-19 crisis, Health risk communication, Social network analysis, Street-level bureaucrats, Willingness to implement policy

## Abstract

Street-level health bureaucrats have actively contributed to implementing the COVID-19 prevention, control, and treatment policies of the Myanmar government. However, the need for bureaucrats on the frontlines of policy implementation to maintain a safe distance from others to prevent the spread of COVID-19 has posed challenges for the sharing and exchange of information related to health risks. In this context, this study examined what health risk communication patterns have emerged and developed among street-level health bureaucrats during the COVID-19 pandemic, and how this risk communication has been affected by street-level health bureaucrats’ perceptions of client meaningfulness and willingness to implement COVID-19 policies. The results reveal that street-level health bureaucrats in the health risk communication network are embedded in reciprocally or transitively connected discussion relationships that sustain their health risk communication over time. Moreover, when specific healthcare staff members perceive more benefits of COVID-19 policies for their patients and are more willing to care for patients, other healthcare staff avoid them to protect themselves from COVID-19 infection. Due to their higher level of understanding of the adopted measures, healthcare staff members who are highly willing to implement COVID-19 policies are frequently approached by other staff members to communicate about COVID-19 issues. This study empirically contributes to the literature on street-level bureaucrats in times of pandemic crisis by examining the formation of health risk communications in the context of street-level health bureaucrats’ responses to and participation in public healthcare policy implementation processes.

## Introduction

Coronavirus disease 2019 (COVID-19) was first recognized in Wuhan City in December 2019. The World Health Organization (WHO) declared a Public Health Emergency of International Concern in January 2020 (WHO 2020a) and confirmed that the emergency had become a pandemic in March 2020. The International Federation of Red Cross and Red Crescent Societies classifies pandemics, including COVID-19, as a natural disaster or hazard (Seddighi 2020). Different countries have adopted differentiated local approaches to respond to the COVID-19 pandemic (OECD 2020). Since December 2019, the Myanmar government has addressed the COVID-19 pandemic crisis (MOHS 2019a) through a national central committee called the Prevention, Control, and Treatment of the COVID-19 Committee (OCHA 2020). During the coronavirus pandemic, this committee has implemented several public health policies, and many public hospitals have treated patients for COVID-19 in a high-risk health setting. Central government orders to maintain social distancing do not imply that the social proximity or connectedness needed to communicate health risks should be sacrificed when street-level bureaucrats deliver public healthcare services to citizens. Street-level bureaucrats in the public sector are frontline public service deliverers who implement upper-level governmental policies and decisions and directly contact their clientele or service recipient groups in a society (Lipsky 1980). These bureaucrats include doctors, nurses, and other healthcare staff who translate the intents of healthcare policies and programs designed by politicians and higher-level public officials into specific outcomes in their local contexts of policy implementation (Lotta and Marques 2020).

Myanmar’s military has also collaborated in the committee’s COVID-19 prevention, control, and treatment measures, with several local military hospitals and street-level military health functionaries offering support (MWD 2020a). Several recent studies have reported the behaviors and psychological, physical, and administrative burdens of street-level health bureaucrats during the pandemic (Lima-Silva et al. 2020; Brunetto et al. 2021; Meza et al. 2021; Van Roekel et al. 2021), but the military healthcare setting has not been investigated, even though military personnel usually live and work within a closed group and are more vulnerable to infectious diseases (Heo et al. 2020). Furthermore, as the Myanmar military has deeply penetrated Myanmar public policy and disaster management (Zaw and Lim 2017; Myomin and Lim 2022), street-level military health bureaucrats are substantially involved in the implementation of COVID-19 policies through the treatment of patients in military hospitals.

While contributing to policy implementation, street-level health bureaucrats have needed to maintain a safe physical distance from any actually or potentially infected patients to prevent the spread of COVID-19. This poses challenges for health risk communication for the purpose of sharing and exchanging medical or public health information not only between bureaucrats but also between bureaucrats and patients. It has become necessary to examine street-level bureaucrats’ problems with these new policies and ways that social connections can be developed to support health risk communication in the pandemic crisis. Furthermore, COVID-19 policies cannot be fully implemented without favorable attitudes on the part of street-level public sector health bureaucrats toward patients and a clear understanding of the aims of the policies they are implementing (Civinskas et al. 2021; Møller 2021; Musheno et al. 2021). This means that it is also necessary to recognize the risk perceptions and behavioral responses of street-level health bureaucrats and evaluate their practices associated with health risk communication that have emerged during the COVID-19 pandemic. This study aimed to answer the following research questions: (1) What health risk communication patterns have emerged and developed among street-level public sector health bureaucrats during the COVID-19 pandemic? (2) How is this risk communication affected by street-level health bureaucrats’ perceptions of client meaningfulness and willingness to implement COVID-19 policies?

## The COVID-19 Pandemic Situation in Myanmar

In Myanmar, the government started to emphasize the seriousness of the COVID-19 crisis from December 2019 (MOHS 2019b). To protect people and prevent the spread of the virus, the Ministry of Health and Sports in Myanmar (MOHS) issued stay-at-home orders, curfews, bans on public gatherings, and closures of public events, entertainment venues, and religious institutions from 28 February 2020 (MOHS 2020a). On 13 March 2020, the Myanmar government reported the first two COVID-19-positive patients in the country, and on 17 March, the MOHS announced a 14-day quarantine policy for incoming travelers from any country. On 30 March, the Office of the President of Myanmar announced an executive order and established the national central committee called the Prevention, Control, and Treatment of the COVID-19 Committee, led by the first vice president. General government hospitals across regions and states have been primarily the ones to receive and treat patients infected with COVID-19. On 17 April the MOHS renewed the quarantine policy and extended the period to 21 days to prevent COVID-19 infection.

**Fig. 1:**
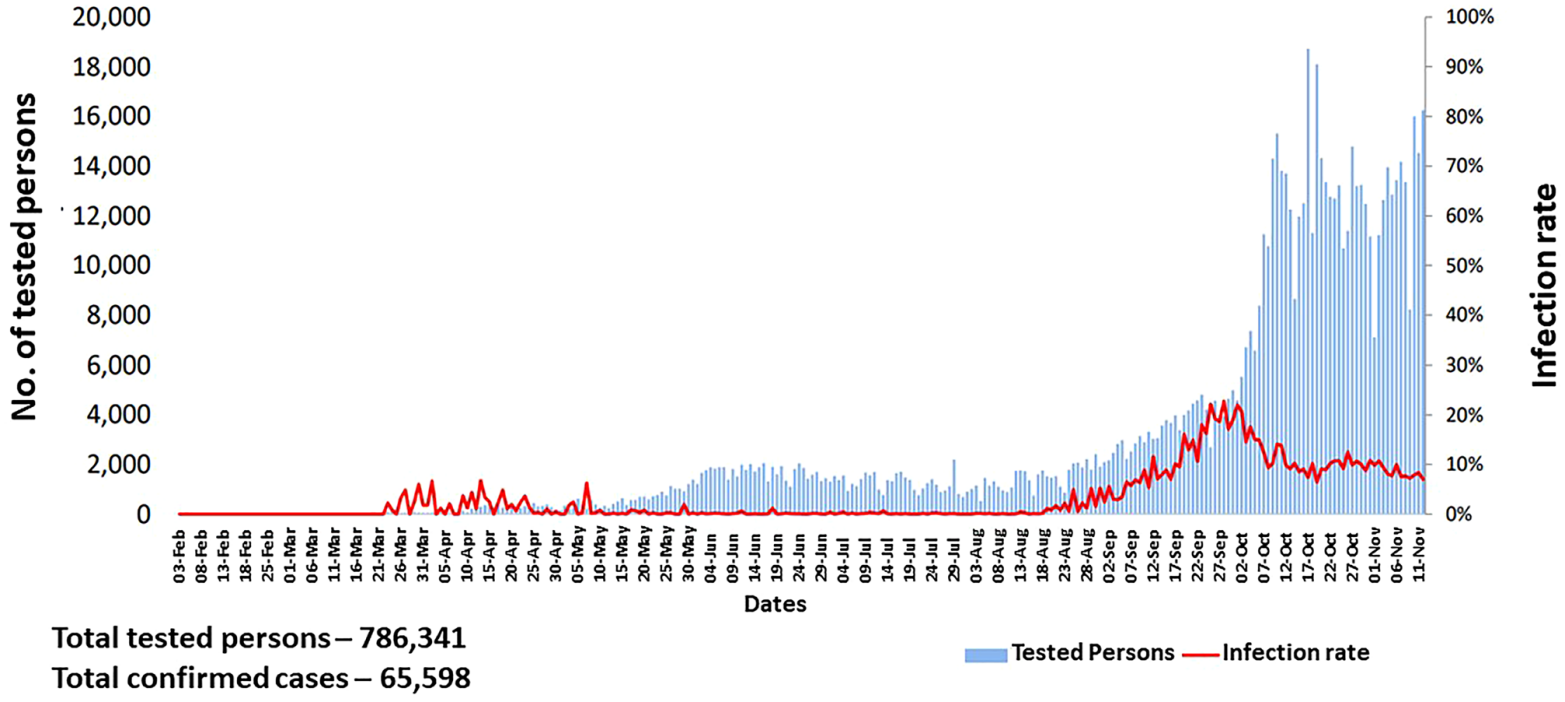
COVID-19 infection rate by date in Myanmar, as of 12 November 2020. *Source* MOHS (2020b).

By June 2020, a total of 16,503 cases had been confirmed across the country, with a rapid increase in daily reported cases and deaths during the reporting period (MOHS 2020b). Thus, the government imposed a nationwide ban on gatherings of 30 or more people, effective beginning 16 August. Moreover, a nationwide curfew from 12:00 a.m. to 4:00 a.m. was imposed, with active government enforcement (U.S. Embassy in Burma 2020). As of 12 November, Myanmar had 65,598 confirmed cases of COVID-19 and 1,508 deaths from the virus, and the infection rate was still high, as shown in Fig. 1.

Throughout the pandemic crisis, the Myanmar military has collaborated with the prevention, control, and treatment initiatives of the COVID-19 committee in the government policy implementation process. In a Myawaddy TV (MWD) broadcast, the Myanmar military’s commander-in-chief said that COVID-19 prevention, control, and treatment are a national security issue. Thus, Myanmar’s military has cooperated with all requirements and has helped the state and citizens through coordinated preparedness and farsighted prevention and treatment initiatives (MWD 2020b). In short, because the military is the most important organization for state security, several local military hospitals have acted in support of COVID-19 prevention, control, and treatment efforts. Accordingly, street-level military health functionaries serving in local military hospitals have actively participated in this process.

## Roles of Street-Level Bureaucrats in Public Healthcare Service Delivery Settings

Most public organizations have street-level bureaucrats, who participate in public policy implementation processes (Hupe and Hill 2007). As the objectives and goals of public policies are ambiguous and street-level bureaucrats have discretion and flexibility in implementing them, their contributions to the implementation process vary (Evans 2010). Bastien (2009) argued that goal ambiguity could increase the level of informal discretion deployed by street-level bureaucrats when the initial level of ambiguity already provides enough flexibility to effectively implement the policy. Thus, ambiguity in street-level bureaucrats’ attitudes could support their informal discretion and lead them to perform differently in the relevant policy implementation process. In healthcare contexts, frontline healthcare staff, as street-level health bureaucrats, have played a critical role in the COVID-19 outbreak response and are the backbone of the country’s safety and public health efforts to contain the spread of disease. They provide the necessary care for patients with suspected and confirmed COVID-19 infections, often under challenging circumstances. They also face a higher risk of infection in their efforts to protect the community (WHO 2020b).

Focusing on street-level bureaucrats’ role, Lipsky (1980) analyzed the behavior of one such type of bureaucrat, namely, frontline staff in public service delivery agencies. Street-level bureaucrats interact directly with citizens or service recipients and have substantial discretion in executing their work. When street-level bureaucrats respond to citizens’ needs and deliver public services to clients, they often face limited resources, such as information, time, and budget (Hupe et al. 2015; Evans 2010). Furthermore, the goals and intents of the public policy that they need to translate into specific outcomes or the rules that they should comply with may not be clear or correspond to local contexts of policy implementation (Lipsky 1980). Consequently, street-level bureaucrats have a substantial degree of discretion in their treatment of their citizens and develop their own coping mechanisms to adaptively respond to diverse citizens’ demands and local circumstances where a certain policy or program is actually carried out (Walker and Gilson 2004). In particular, the severe challenges that the ongoing unprecedented COVID-19 pandemic situation poses for street-level health bureaucrats—including increased demand for healthcare services, insufficient resources, and a lack of scientific knowledge and the resultant ambiguity—grant more discretion autonomy to them (Dunlop et al. 2020; Lima-Silva et al. 2020; Carlitz et al. 2021; Davidovitz et al. 2021; Mojica Méndez et al. 2021). Therefore, their attitudes toward patients and efforts to decipher policy directions from policymakers clearly become more critical for implementing COVID-19 policies and achieving policy goals.

Tummers and Bekkers (2014) examined the discretion and autonomy of street-level bureaucrats in policy implementation. They presented the concept of client meaningfulness, which can be defined as street-level bureaucrats’ perception that carrying out a public policy is valuable for their target clients or service recipients. For instance, medical staff might experience client meaningfulness when they implement a policy focused on helping their patients get well. According to this argument, street-level bureaucrats aim to implement policies and try to make them work conditional on client meaningfulness levels (Tummers and Bekkers 2014). In addition, Metselaar (1997) argued that discretion exerted by street-level bureaucrats could positively affect their willingness to implement policy. Willingness to implement policy could be defined as a street-level bureaucrat’s positive behavioral intention to implement a public policy (Ajzen 1991; Metselaar 1997). In particular, in the healthcare environment, street-level bureaucrats could tailor their decisions and the procedures that they follow to their clients’ specific situations and needs, which has significantly different consequences for performance and risk management in implementing health policies (Tummers and Bekkers 2014). Thus, risk communication among street-level health bureaucrats can be affected by their willingness to implement policies and respond to their clientele’s demands. More specifically, the exchange of health information and discussion among street-level health bureaucrats may be related to their willingness to implement policies and consider patients’ needs and situations.

## Street-Level Health Bureaucrats in Times of Pandemic Crisis

Typically, street-level bureaucrats have intrinsic motivations, which are essential to supporting social well-being (Lynn et al. 2001), and interact with citizens directly in the process of public service provision. One of the key roles of street-level bureaucrats is to process vast information on citizens’ competing desires and requests during public service delivery (Prottas 1979). Gofen and Lotta (2021) indicated that street-level implementation and crisis are not disjointed concepts. A crisis generally increases the need for public services provided by different street-level bureaucrats while disrupting their ordinary day-to-day practices or knowhow and requiring reformulation of street-level implementation activities. Obviously, during a crisis, street-level bureaucrats experience greater and unexpected demand for critical public services, accompanied by a lack of resources to meet citizens’ urgent demands (Alcadipani et al. 2020; Dunlop et al. 2020). In this context, the additional assigned duties beyond the previous day-to-day practices of street-level bureaucrats reflect three interrelated changes in their policy implementation: policy ambiguity, expanded discretion, and greater risk exposure (Davidovitz et al. 2021). More frequent issuance of official orders during a crisis leads to an environment of increased policy ambiguity. The consequent situational flexibility expands discretion in street-level implementation, in interaction with street-level bureaucrats’ willingness to take greater risks in times of pandemic crisis (Davidovitz et al. 2021).

Lipsky (1980) saw healthcare workers as street-level bureaucrats in the traditional sense, but street-level health bureaucrats are strongly affected by their own professional identities (Harrits and Møller 2014). Street-level health bureaucrats must mediate between two distinct operations: the effort to implement policy according to rules and the effort to apply and adjust rules to specific and concrete cases (Bannink et al. 2015). Some evidence also indicates that working under emergency conditions or during extreme crises raises risks for street-level health bureaucrats (Paton 2006), including risks of stress, depression, and other psychological disorders (Sifaki-Pistolla et al. 2017; Mao et al. 2018).

In the context of the COVID-19 pandemic crisis, street-level health bureaucrats concerned with mediating and balancing their roles and positions to deal with the critical situation and to process client needs have been adequately supported in their crucial decision making regarding the health and lives of current or prospective patients (Meza et al. 2021). In the face of high levels of uncertainty, street-level health bureaucrats’ professional identity firmly guides their decision making, communications, and continuous professionalization as an effective coping mechanism (Gofen et al. 2021; Meza et al. 2021). On the other hand, due to limited resources and increased risks on the ground, street-level health bureaucrats may resist implementation of new policies and express frustration with worsening work conditions (Cox et al. 2021).

In a pandemic situation, governments relax their rules, which might place additional burdens on their street-level bureaucrats, who already suffer from a lack of relevant information, limited resources, heavy workloads, and policy ambiguity. Thus, crises might change street-level bureaucrats’ roles and influence their operations. Additionally, street-level bureaucrats can use discretion to prioritize the needs and requirements of customers (Collins and Augsberger 2021). On the other hand, efforts by organizations to limit street-level bureaucrats’ discretion in their interactions with clients may affect these bureaucrats’ status and roles (Pérez-Chiqués et al. 2021).

## Health Risk Communication Networks Among Street-Level Health Bureaucrats in the Pandemic Crisis: Hypothesis Development

Many health communication concepts describe the transfer of information from one source to another, which could be conceived of as connections in a network. On the other hand, health risk communication can be defined as an interactive process of exchanging information and opinions among individuals in a community concerning a potential risk to human health or the environment to help them make better decisions about their well-being during a crisis (Lim et al. 2016). Health-related behavioral changes may be transmitted through each actor’s connections with other actors by means of their deliberations about certain health risks (Dao and Lim 2022). For example, Lim and Nakazato (2020) revealed the role of social connectedness in risk information-sharing in a community during the global COVID-19 pandemic and reported that selection and social influence mechanisms coexist, affecting each citizen’s health-related behaviors in the health crisis. Additionally, Zhang et al. (2020) argued that delayed decision making and limited information disclosure by government officials significantly influence the effectiveness of health risk communication.

**Fig. 2:**
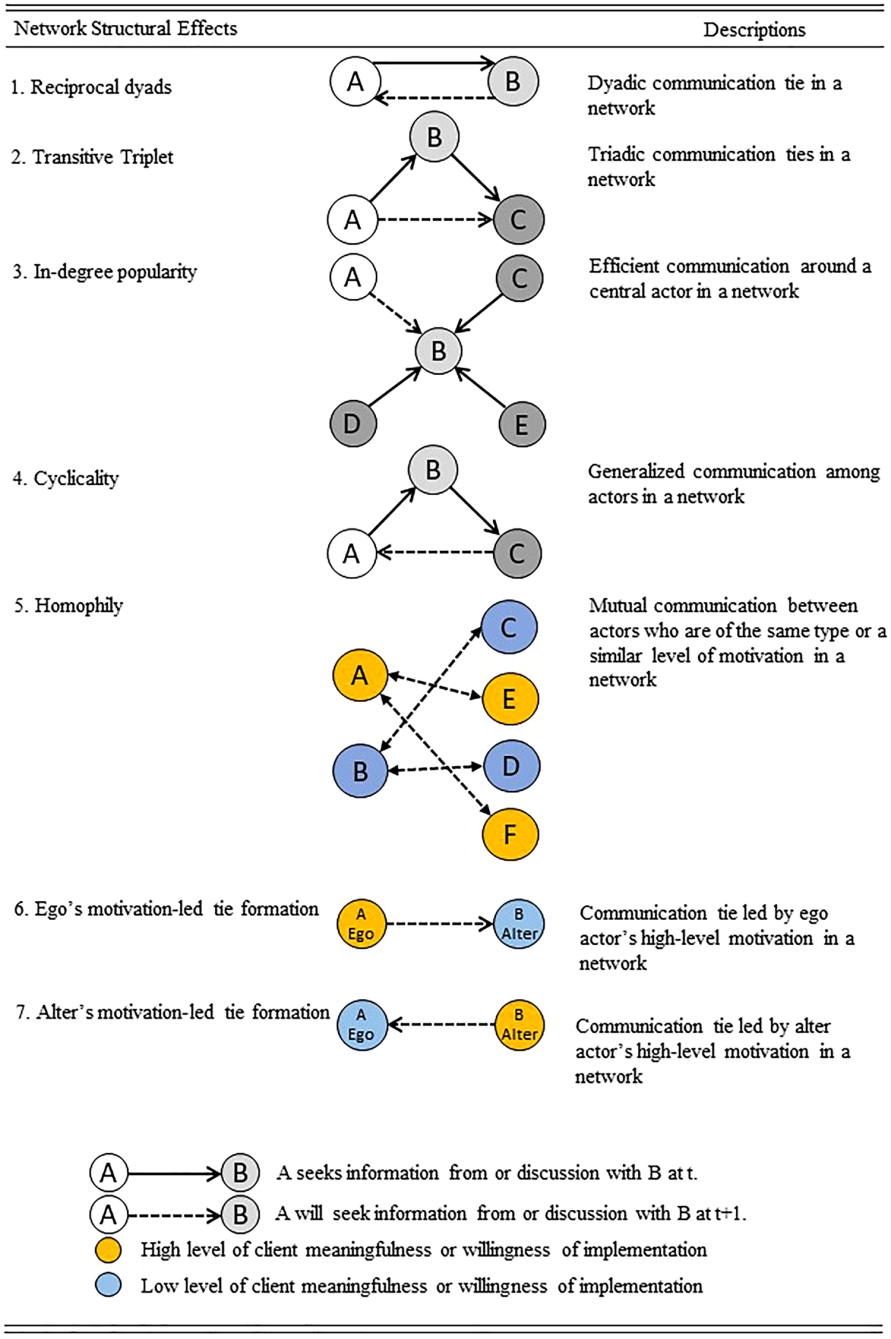
The hypothesized network structural effects

Theoretically and methodologically, social network concepts and analyses are increasingly leveraged for healthcare analytics, especially in relation to risk communication, disease prevention, and the study of healthcare organizations and systems (Levy and Pescosolido 2002; Lee et al. 2016). The primary way to sustain trustworthiness and credibility in a risk communication network system is through two types of closely connected network structures: reciprocal relationships and transitive relationships (Nakazato and Lim 2016; Lim and Nakazato 2019). The formation of reciprocal bonding ties between actors in the communication process can enhance credibility through mutual deterrence (Nakazato and Lim 2017; Lim and Nakazato 2020). Such reciprocal relationships can cultivate trust between actors, which is vital in facilitating communication processes because they can directly share health risk information with each other in turn. Therefore, reciprocal dyads are used to measure this type of mutual communication relationship in a network (see Fig. 2, network structural effect 1).

H1 (Reciprocal Dyads): *In a health communication network, when street-level health bureaucrat A initiates communication or discussion about COVID-19 issues with street-level health bureaucrat B, B will tend to communicate/interact with A in the future.*

Actors also tend to form relationships with their partner’s partner to discuss health risk information. The emergence of such a transitive triplet could foster trustworthiness and credible commitments among triadic actors in a risk communication network, leading to the formation of network clusters and tightly connected subgroups. Triadic ties are denser than dyadic ties and constitute a more closed network in which health information can be more easily double-checked and monitored by being circulated through communication and discussion (Nakazato and Lim 2017; Lim and Nakazato 2020) (see Fig. 2, network structural effect 2).

H2 (Transitivity): *In a health communication network, when street-level health bureaucrat A initiates communication or discussion about the COVID-19 issue with street-level health bureaucrat B and B initiates communication or discussion with street-level health bureaucrat C, then A will be likely to communicate about or discuss the COVID-19 issue with C (that is, the previous partner (B)’s partner) in the future*.

Some actors possessing higher amounts of information may become increasingly popular among network actors (Granovetter 1985; Burt 1992). Actors in a risk communication network are more likely to seek reliable communication partners located at the center of their dialogue network (Berardo and Scholz 2010). To measure this type of tendency, in-degree popularity is used (see Fig. 2, network structural effect 3).

H3 (In-Degree Popularity): *In a health communication network, when street-level health bureaucrat B receives requests to communicate about or discuss the COVID-19 issue from many actors, including C, D, E, or others, and his/her reputation spreads, street-level health bureaucrat A will also have a greater likelihood of seeking information about COVID-19 issues from B*.

If some actors in a risk communication network receive requests to discuss current health risk issues too frequently, the communication burden could become concentrated around these actors. To resolve such an accumulation of indebtedness among actors, cyclical triads for the generalized exchange of information could be formed, and the information-sharing burden could be spread among them (Molm and Cook 1995; Lim and Nakazato 2020). That is, actors may communicate and share information with one another without expecting a return directly from the specific actors who requested information and with whom they were linked before (see Fig. 2, network structural effect 4).

H4 (Cyclicality): *In a health communication network, when street-level health bureaucrat A communicates about the COVID-19 issue with street-level health bureaucrat B and B also communicates with street-level health bureaucrat C, C will subsequently tend to communicate with A*.

Homophily arises when the formation of ties is initiated based on similar individual attributes or the same characteristics between actors (McPherson et al. 2001; Wukich et al. 2019; Lim and Nakazato 2020). The selection of partners with whom to discuss and communicate about health risks based on similarities in street-level health bureaucrats’ individual properties, such as job type (that is, doctors, nurses, and other medical staff) and tenure, could reduce transaction costs related to searching for discussion partners and controlling the quality of information exchanged and enhance the effectiveness of deliberation in health risk communication processes (see Fig. 2, network structural effect 5).

H5 (Homophily in Street-Level Health Bureaucrats’ Type or Tenure): *Street-level health bureaucrats are more likely to forge ties for discussion with other street-level health bureaucrats of the same job type or similar tenure in a health communication network*.

Street-level health bureaucrats’ perceptions regarding client meaningfulness—that is, their insight and understanding that a policy that they implement is critical for improving the quality of lives of their clients—have an absolute value for their clients. For instance, medical staff might experience client meaningfulness when implementing a policy concentrated on helping patients get well (Tummers and Bekkers 2014). Thus, street-level health bureaucrats with some level of client meaningfulness may initiate ties with other street-level health bureaucrats who have a similar level of client meaningfulness in a health risk communication network. Furthermore, similar levels of willingness among street-level health bureaucrats to implement new governmental policies to address COVID-19 pandemic risk (Tummers 2011; Tummers et al. 2012) could also be a reason for their selection of each other as discussion partners.

H6 (Homophily in Client Meaningfulness): *Street-level health bureaucrats are more likely to establish discussion ties with others having a similar level of client meaningfulness in a health communication network*.

H7 (Homophily in Willingness to Implement Policy): *Street-level health bureaucrats are more likely to establish partnerships with those in the health communication network who have a similar willingness to implement policy*.

Furthermore, street-level health bureaucrats could have different impacts on their clients’ lives when implementing a policy based on the ego’s and alter (partner)’s differential motivation levels (Maynard-Moody and Musheno 2000).^1^ In the context of the COVID-19 pandemic, street-level health bureaucrats themselves are highly exposed to the risk of COVID-19 infection in their risky working places, and their workloads under the uncertain pandemic emergency setting have increased greatly (Civinskas et al. 2021; Davidovitz et al. 2021; Musheno et al. 2021). Their patients are also isolated from regular healthcare services, as they are protected from physical interactions in hospitals (Møller 2021). Furthermore, if some medical staff are willing to care more for patients, they may avoid or be avoided by other healthcare staff to minimize potential exposure (Brunetto et al. 2021). Nevertheless, in the course of performing their duties and fulfilling their responsibilities, healthcare street-level health bureaucrats must communicate with other medical staff to provide timely and effective support to their patients. Thus, when the client (patient) meaningfulness of the ego or alter (partner) is higher (that is, if healthcare street-level health bureaucrats strongly perceive that implementing COVID-19-related policies is critical to their patients’ health and well-being), the bureaucrats could be more likely to seek information about current health risk issues through communication with other street-level health bureaucrats to more adequately respond to their patients’ needs.

H8 (Ego’s Client Meaningfulness as a Tie Initiator): *When the client (patient) meaningfulness of the ego in a street-level health bureaucrat’s network is higher, a tie for risk communication is more likely to be established among street-level health bureaucrats*.

H9 (Alter’s Client Meaningfulness as a Tie Initiator): *When a partner street-level health bureaucrat’s level of client meaningfulness is higher, a tie for risk communication is more likely to be established among street-level health bureaucrats*.

When an ego or partner healthcare street-level health bureaucrat is more acquiescent with governmental pandemic response guidance and more willing to implement the decisions made by governmental health authorities (Tummers 2011; Tummers et al. 2012), a tie is more likely to be initiated by these bureaucrats to collect more relevant information about health risks. Thus, the ego’s or partner’s willingness to implement COVID-related policies could affect tie initiation in the health risk communication network based on the node’s level of inspiration or motivation.

H10 (Ego’s Willingness to Implement Policy as a Tie Initiator): *When the level of willingness to implement policy of the ego in a street-level health bureaucrat’s network is higher, a tie for risk communication is more likely to be established among street-level health bureaucrats*.

H11 (Alter’s Willingness to Implement Policy as a Tie Initiator): *When the level of willingness to implement policy of a partner street-level health bureaucrat is higher, a tie for risk communication is more likely to be established among street-level health bureaucrats*.

## Methodology

The empirical analysis of this study is based on a survey of health risk communication networks formed in a local military hospital in Myanmar with 100 beds and various street-level military medical bureaucrats (doctors, nurses, and other medical staff) working together to provide COVID-19 pandemic crisis response.^2^ This social network analysis survey (Adams 2020) was distributed in this local military hospital, and data were collected during three time periods: 27 to 30 September, 6 to 9 October, and 16 to 19 October, 2020. Longitudinal data collection was chosen because previous research on communication networks employing cross-sectional data has been limited in its ability to analyze the dynamic nature of the communication relationships among actors and the antecedents of their tie formations for communication (Monge and Contractor 2003; Contractor et al. 2012). In particular, appropriately understanding the emergence and evolution of risk communication networks and the underlying mechanisms behind these network dynamics during a crisis or disaster requires the collection of a panel dataset of risk communication observed among the same set of network actors across time (Lim and Nakazato 2020; Dao and Lim 2022).

Responses were received from all 50 street-level military health bureaucrats in this hospital; the medical staff were queried on their health risk communication with other staff in this hospital, role perceptions and motivations as street-level bureaucrats, and personal backgrounds. All respondents were male because the military hospital is dominated by males. On average, the respondents included in the analysis had worked 4.44 years for this hospital. Most of the respondents were either medical doctors (26%) or nurses (62%) (see Table 1). Questionnaires were completed with various survey methods according to each military medical staff member’s preference: face to face, via telephone, or online. As our access to some medical staff was limited due to the ongoing pandemic crisis, this flexible data collection method allowed the respondents to choose which survey method worked for them.

**Table 1:** Characteristics of participants in the survey (*N* = 50)

Demographics	Mean (S.D.) or percentage	Max.	Min
Gender	Male: 100%; female: 0%		
Working years	4.44 (4.17)	21	1
Job	Medical doctors: 26%; nurses: 62%; other staff: 12%		

The social network survey questionnaire used in this study mainly asked all 50 respondents to report their interactions for COVID-19-related risk communication with other actors at the same hospital (“Among the 50 medical staff on the list, whom did you approach to discuss recent coronavirus issues over the last week? Select as many as possible out of the 50 medical staff in your hospital.”). The same list of 50 street-level military health bureaucrats in the survey questionnaire was shown to these 50 respondents to report their communication partners during each time period.^3^ Therefore, the dataset presents individual street-level health bureaucrats’ ties with other staff for health risk communication and discussions, transformed into actor-by-actor (50 × 50) binary adjacency matrices in which the cells show (0) the absence or (1) the presence of communication. In addition to this collection of the social network data on risk communication, we conducted semistructured interviews with 11 of the 50 respondents selected at random. We asked two open-ended questions: “Could you describe how the COVID-19 situation has altered your work in your hospital?” and “With whom did you discuss COVID-19 problems, and how?” The qualitative data collected from these interviews supplemented the main social network data, as the former helped us interpret, contextualize, and triangulate the results of social network analysis based on the latter (Yousefi Nooraie et al. 2020).

In order to capture bureaucrats’ motivations and behaviors in carrying out the policies and programs for COVID-19 response, measures of client (patient) meaningfulness (five survey items) and willingness to implement policy (four survey items) were adapted from Tummers and Bekkers (2014) with Likert-type five-point scales and used in the analysis by taking each actor’s mean values for these two concepts.

With respect to methodology, we employed stochastic actor-oriented modeling (SAOM) using a statistical tool—the simulation investigation for empirical network analysis (SIENA) (Ripley et al. 2022)—to analyze the evolution of health risk communication among the 50 frontline healthcare workers over time. Stochastic actor-oriented modeling is appropriate for analyzing longitudinal network dynamics in terms of individual actors’ decisions to change their tie formation status, including forging, terminating, or maintaining ties (Lim and Nakazato 2020; Aung and Lim 2021; Lim 2021). In particular, SAOM helps us model the longitudinal changes in risk communication networks as emergent properties from the aggregates of individual actors’ choices of communication partners, which will mutually limit and enable each other’s choices across time periods within the networks (Contractor et al. 2012; Lim and Nakazato 2020).

## Results

**Fig. 3:**
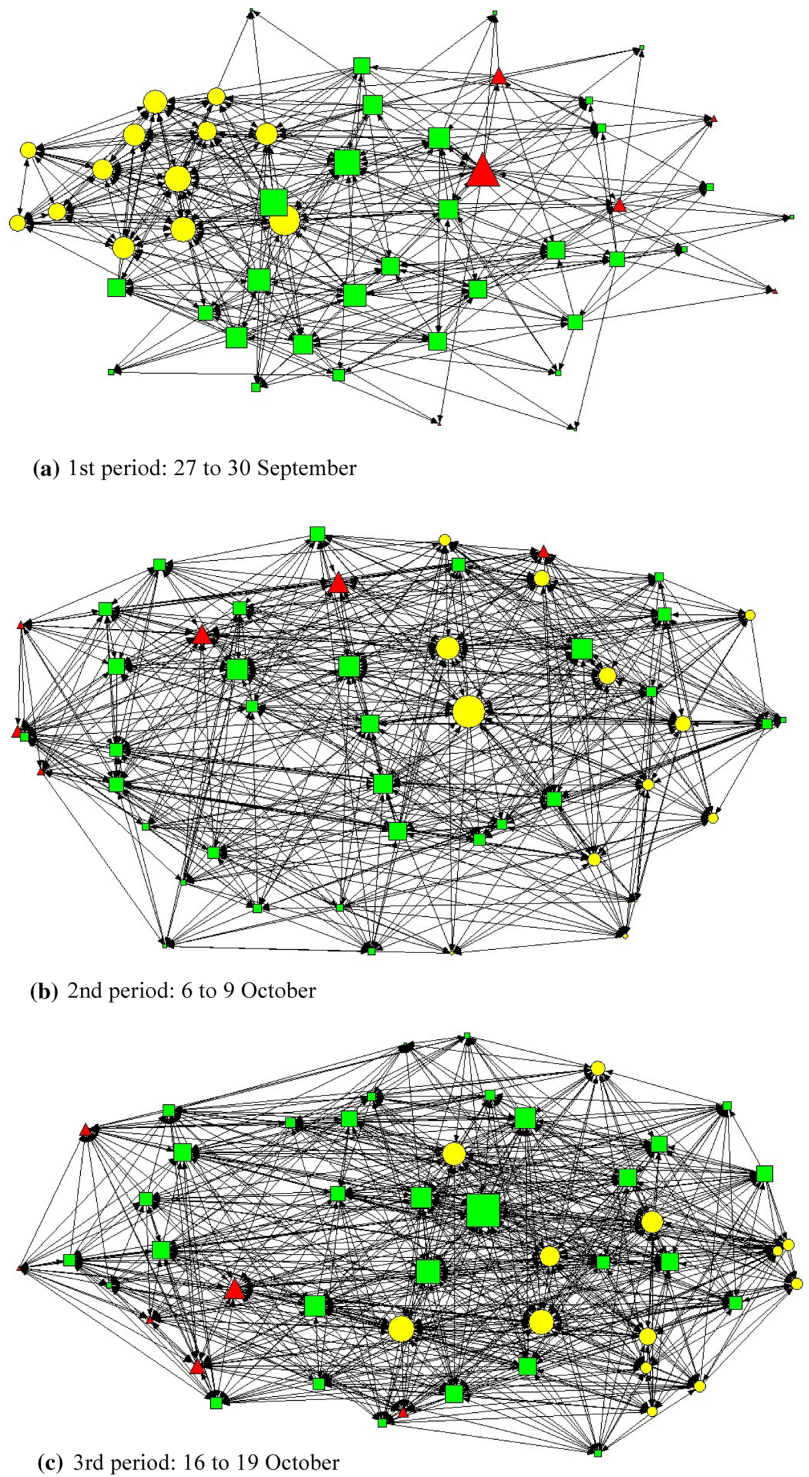
Health risk communication ties among street-level health bureaucrats in the COVID-19 policy implementation network. Arrowheads indicate street-level health bureaucrats and illustrate the directions of health risk communication or discussion among them. Arrows start from health risk information seekers or requesters for discussions of COVID-19 issues. The shapes show the types of street-level health bureaucrats: yellow circles for doctors, green squares for nurses, and red triangles for other medical staff. The size of the symbols is proportional to each street-level health bureaucrat’s degree centrality.

Figure 3 shows the sociograms for the emergence and development of ties for health risk communication and discussion activities among the 50 military health bureaucrats over three different time periods in the COVID-19 pandemic crisis.

Table 2 presents the estimated results on the formation of ties in the health risk communication network. First, in the model of network dynamics exploring how densely connected strong ties are developed, the results supported that actors in the health communication network tended to form reciprocal dyadic ties (Hypothesis 1) (parameter 4: 0.13, *p* < 0.10). The bureaucrats were more likely to communicate about health risk issues and COVID-19 prevention policies with other bureaucrats in the same local military hospital with whom they had already communicated. One medical doctor interviewed confirmed this reciprocal communication: “I mostly contacted a number of medical staff, particularly those who had previously informed me of hospital issues, patients’ issues, COVID-19 issues and policies at the time.” The formation of links for health risk communication was also driven by transitivity (Hypothesis 2) (parameter 5: 0.08, *p* < 0.01), which means that the bureaucrats tended to initiate communication with or seek information from former discussion partners’ partners. One nurse and one doctor also mentioned this triadic relationship for communication in the interviews: “In general, when conducting my job in the COVID-19 pandemic situation, I mostly communicated with my close coworkers and coworkers’ friends to lessen the risks”; “In that time, I spoke with a number of medical staff, including not only those who had previously informed me of COVID-19 difficulties and logistics but also some medical personnel who discussed with those.”

**Table 2:** Estimated results for the formation of ties in the health risk communication network among street-level health bureaucrats

Variables	Coefficients	Standard error
1. Rate of change from 1st period to 2nd period	34.82^***^	3.07
2. Rate of change from 2nd period to 3rd period	3.71^***^	0.31
3. Out-degree (density)	− 0.69^***^	0.22
4. Reciprocal dyads	0.13^*^	0.07
5. Transitivity	0.08^***^	0.01
6. In-degree popularity (square root)	− 0.09	0.06
7. Cyclicality	− 0.02	0.01
8. Homophily in street-level health bureaucrats’ type	0.07	0.06
9. Homophily in street-level health bureaucrats’ tenure	0.01	0.12
10. Homophily in client meaningfulness	0.50^***^	0.19
11. Ego’s client meaningfulness as a tie initiator	0.09^*^	0.05
12. Alter’s client meaningfulness as a tie initiator	− 0.12^**^	0.05
13. Homophily in willingness to implement policy	0.16	0.17
14. Ego’s willingness to implement policy as a tie initiator	− 0.004	0.03
15. Alter’s willingness to implement policy as a tie initiator	0.08^**^	0.03

***Significant at the 1% level; **Significant at the 5% level; *Significant at the 10% level

The coefficients are from a standard simulation investigation for empirical network analysis (SIENA) of a directed network with 50 military medical staff across three time points. The overall maximum convergence ratio is 0.19, and all statistics converge with t-ratios close to zero (< 0.10) with a minimum of 3000 iterations

This study predicted that the bureaucrats would be more likely to communicate about or discuss COVID-19 issues with popular bureaucrats as the reputation of the latter as central actors within the network spreads (Hypothesis 3). However, the bureaucrats in this health risk communication network did not rely heavily on popular bureaucrats or regard them as reliable communication partners, as shown by the statistically nonsignificant effect of in-degree popularity (parameter 6). In the context of the pandemic emergency, popular actors in this risk communication network could be regarded as potential transmitters of COVID-19 rather than efficient coordinators or disseminators of information, which was indicated by one nurse in the interview: “There is one person in our group who works hard. So, he has more and more contact with patients and health workers on a daily basis. Admittedly, I kept in touch with him as little as possible because of my fears that he might be infected. In particular, I made an effort to approach fewer medical staff members who were already being contacted by a large number of medical staff members and patients as a result of their status and position.” Moreover, in the study, there was no homophily relationship between street-level health bureaucrats (Hypothesis 5) of the same job type (parameter 8; for example, between military medical doctors, between nurses, or between other medical staff) or similar tenure (parameter 9) because regardless of their task types or service years, all medical staff members’ duties became uncertain and riskier during the pandemic crisis. This was pointed out by one medical doctor and one general medical staff member in the interviews: “During the pandemic, we did not strictly separate our roles such as doctors and nurses”; “Even though we were in different roles as doctors, nurses, and medical staff with seniority, we worked together when we were serving in the hospital because we only had a limited number of human resources and time available.”

Concerning the effects of homophily in the selection of health communication partners based on the bureaucrats’ policy implementation motivations (that is, client meaningfulness (Hypothesis 6) and willingness to implement policy (Hypothesis 7)), a homophily effect was found based only on client meaningfulness (parameter 10: 0.50, *p* < 0.01), supporting Hypothesis 6. That is, there were more risk communication interactions between healthcare staff with a similar client meaningfulness level, as supported by one medical doctor we interviewed: “In spite of my own psychological discomfort, I am willing to communicate with the feisty medical staff because we must work together to implement the COVID-19 policies and save patients’ lives. Furthermore, I maintained regular communication with my coworkers who had a favorable opinion of the COVID-19 regulations and who considered the policies beneficial to their own clients.”

Furthermore, the client meaningfulness of the ego in the bureaucrats’ network served as a tie initiator (parameter 11: 0.09, *p* < 0.10). When a bureaucrat’s level of client meaningfulness is higher than that of a potential communication partner, the staff member with a higher level of client meaningfulness is more likely to actively seek and exchange information about current health risk issues through communication with other medical staff to carry out COVID-19-related policies for patients more appropriately and in a timelier manner, as shown in one medical doctor’s narrative: “I talked to my nurses on a daily basis that we must strictly adhere to our COVID-19 policies to benefit our patients and that we must also exercise greater caution in our contact with patients as well as with our coworkers in the same hospital.” In contrast, when a partner bureaucrat’s degree of client meaningfulness is higher than that of the ego, a tie for risk communication was less likely to be established between these staff members (parameter 12: − 0.12, *p* < 0.05). Partners’ higher client meaningfulness implies more frequent contact with patients to share the potential benefits of COVID-19-related measures for health and safety and adapt these measures to their patients’ specific situations by using their discretion on the grounds of policy implementation (Tummers and Bekkers 2014). As a result, these bureaucrats have a higher risk of being infected by COVID-19 in the pandemic situation, and the client meaningfulness of the alter did not serve as a tie initiator in this risk communication network. More specifically, healthcare staff who were more willing to care for their own patients and protect the patients from the pandemic seem to have been avoided by other staff to avoid infection in the pandemic crisis, as one nurse interviewed confirmed: “Despite their optimistic views of the benefits of implementing the COVID-19 policies for their patients in this military hospital, some coworkers have been infected with the COVID-19 virus due to their frequent interactions with patients. Such a worry makes me avoid being a primary contact with them.”

Conversely, an alter’s willingness to implement new COVID-19-related policies or programs did trigger tie formation (parameter 15: 0.08, *p* < 0.05). When a partner bureaucrat’s level of willingness to implement policy is higher than that of the ego, a tie for risk communication is more likely to be forged. When partner healthcare staff are more compliant with governmental guidance on pandemic response and willing to implement new and urgent decisions made by governmental health authorities, their approaches are more likely to be accepted by other staff to communicate about and discuss COVID-19 issues due to the receptiveness of the former to governmental directions and potentially their greater understanding of the objectives and goals of the measures adopted by the government and assigned for implementation. Such an influence of highly motivated alters’ approaches is evident in the following statements by one medical doctor and one nurse: “Fortunately, as a result of the support of and discussion with my coworkers, I am able to deal with the difficulties I confront at work. My mental strength is boosted by the advice and support from my coworkers, as well as my faith in God, which allows me to remain optimistic in the face of adversity”; “I acknowledged that I was an unwilling nurse at the time. However, with the encouragement of my coworkers who had a higher level of confidence and willingness, I accomplished my jobs effectively, and I always respect them.”

## Discussion

This study focused on how health risk communication ties emerged and formed among street-level military health bureaucrats in their implementation of policies and programs to prevent and control COVID-19 in a Myanmar military hospital. According to the empirical results, the bureaucrats in the examined health risk network were embedded in closely—reciprocally or transitively—connected discussion and communication relationships that resiliently sustained health risk communication. The study found no homophilic relationships or segregated interactions dependent on staff types—for example, between medical doctors only or nurses only—because all medical staff duties were similar in the face of the increased uncertainty during a pandemic caused by a poorly understood virus. Thus, among the military hospital bureaucrats, doctors, nurses, and other staff were not more likely to establish communication partnerships based on health bureaucrat type in the emergent health communication network. Moreover, healthcare staff members who were more willing to care for patients were avoided by other healthcare staff to prevent infection during the pandemic. Although street-level health bureaucrats played a critical role in addressing the outbreak, they also needed to cope with exposure to hazards and protect themselves from the higher risks of COVID-19 infection. On the other hand, due to their greater understanding of the adopted measures, healthcare staff members who were highly willing to implement COVID-19 policies more frequently approached other staff members to communicate about COVID-19 issues and provide necessary information.

The COVID-19 pandemic response has faced many constraints and limitations on public health service delivery, including social distancing measures and the resultant lack of health risk communication. The lessons on health risk communication from a Myanmar public healthcare setting suggest that risk communication strategies for dealing with uncertainties in public healthcare service provision should be improved appropriately. The COVID-19 pandemic has shown that no one is safe until everyone is safe, and thus, the responsibility for risk communication through direct contact with actual and potential patients should be balanced and shared among street-level health bureaucrats. That is, different healthcare actors must fulfill their responsibility according to their roles and keep health communication networks working effectively (Davidovitz et al. 2021). Generally, as patients may lack the intricate professional knowledge needed to understand health risks, healthcare staff are responsible for translating this knowledge into explicit and straightforward content that can be easily understood. This could support health risk communication practitioners and policymakers in developing countries where street-level bureaucrats must frequently adjust their understanding of policy objectives and goals. In particular, during the COVID-19 pandemic crisis, street-level health bureaucrats have experienced greater policy ambiguity and risk. Therefore, it has been necessary for them to find ways to sustain their client meaningfulness, discretion, and willingness to implement policy during the pandemic crisis.

This study empirically contributed to the literature on street-level bureaucrats by examining health risk communications in street-level health bureaucrats’ responses to and participation in public healthcare policy implementation processes (Dunlop et al. 2020). To ensure effective emergency pandemic response, governments need to formulate and mandate appropriate public healthcare service delivery arrangements and responsibilities for street-level health bureaucrats and design incentives for them to act independently where appropriate. This is because street-level public health bureaucrats are close to citizens and best positioned to form the first line of defense against a pandemic. This study also investigated public policy implementation on the ground from a street-level bureaucrat’s perspective and drew lessons on how to think constructively about such bureaucrats’ roles, motivations, and behaviors. In pandemics, healthcare policies also need to consider the morale and socioeconomic safety of street-level health bureaucrats (Gofen and Lotta 2021). The findings suggest that not only bureaucrats’ own but also their colleagues’ levels of client meaningfulness or willingness to implement policy affect their efforts to collect information about ongoing pandemic health risks and understand the aims of the policies and programs that they are implementing in the context of public healthcare service provision. Thus, the study draws attention to the importance of understanding the dynamics of health risk communication and street-level bureaucrats’ behaviors and motivations for policy implementation in the context of the pandemic.

## Conclusion

This study provides a better understanding of street-level health bureaucrats’ motivations, response activities, and actual practice observed in a local military hospital in Myanmar. It focuses on these bureaucrats’ difficulties in implementing new policies and characterizes their emergent ties for health risk communication during the COVID-19 pandemic. In view of the findings, the government should recognize this essential function of public healthcare service workers and the significance of protecting the public healthcare workforce, supporting street-level health bureaucrats’ perceptions of client meaningfulness and willingness to implement COVID-19 policies and programs.

## Footnotes

1 “Ego” is an individual “focal” actor in a network. “Alters” are other individual actors to whom the ego is tied in the same network (Borgatti and Halgin 2011).

2 Although the present study empirically demonstrates how health risk communication can be effectively used for healthcare service delivery and health policy implementation, there are some limitations that need to be considered seriously in future research. First, this research approach could be expanded to assess risk communications under other emergency settings with different scales and scopes and connected to other policy environments where street-level bureaucrats are embedded. Second, this study suggests further work on how health risk communication could be related to the effectiveness and sustainability of public healthcare policy implementation.

3 Out of the three data collection methods—face to face, via telephone, or online, the respondents answering using the two former methods frequently confirmed their understanding of the survey items directly with the authors. This might have improved the validity and reliability of the data collected by these methods compared to the data collected using an online tool (Adams 2020).

## References

[CR1] Adams, J. 2020. *Gathering social network data*. Thousand Oaks, CA: Sage.

[CR2] Ajzen, I. 1991. The theory of planned behavior. *Organizational Behavior and Human Decision Processes* 50(2): 179–211.

[CR3] Alcadipani, R., S. Cabral, A. Fernandes, and G. Lotta. 2020. Street-level bureaucrats under COVID-19: Police officers’ responses in constrained settings. *Administrative Theory & Praxis* 42(3): 394–403.

[CR4] Aung, T.M., and S. Lim. 2021. Evolution of collaborative governance in the 2015, 2016, and 2018 Myanmar flood disaster responses: A longitudinal approach to a network analysis. *International Journal of Disaster Risk Science* 12(2): 267–280.

[CR5] Bannink, D., F. Six, and E. van Wijk. 2015. Bureaucratic, market or professional control? A theory on the relation between street-level task characteristics and the feasibility of control mechanisms. In *Understanding street-level bureaucracy*, ed. P. Hupe, M. Hill, and A. Buffat, 205–226. Bristol: Policy Press.

[CR6] Bastien, J. 2009. Goal ambiguity and informal discretion in the implementation of public policies: The case of Spanish immigration policy. *International Review of Administrative Sciences* 75(4): 665–685.

[CR7] Berardo, R., and J.T. Scholz. 2010. Self-organizing policy networks: Risk, partner selection, and co-operation in estuaries. *American Journal of Political Science* 54(3): 632–649.

[CR8] Borgatti, S.P., and D.S. Halgin. 2011. On network theory. *Organization Science* 22(5): 1121–1367.

[CR9] Brunetto, Y., N. Saheli, T. Dick, and S. Nelson. 2021. Psychosocial safety climate, psychological capital, healthcare SLBs’ wellbeing and innovative behaviour during the COVID 19 pandemic. *Public Performance & Management Review*. 10.1080/15309576.2021.1918189.

[CR10] Burt, R.S. 1992. *Structural holes: The social structure of competition*. Cambridge, MA: Harvard University Press.

[CR11] Carlitz, R., T. Yamanis, and H. Mollel. 2021. Coping with denialism: How street-level bureaucrats adapted and responded to COVID-19 in Tanzania. *Journal of Health Politics, Policy and Law* 46(6): 989–1017. 10.1215/03616878-9349128 34075413

[CR12] Civinskas, R., J. Dvorak, and G. Šumskas. 2021. Beyond the front-line: The coping strategies and discretion of Lithuanian street-level bureaucracy during COVID-19. *Corvinus Journal of Sociology and Social Policy* 12(1): 3–28.

[CR13] Collins, M.E., and A. Augsberger. 2021. Impacts of policy changes on care-leaving workers in a time of coronavirus: Comparative analysis of discretion and constraints. *Journal of Comparative Policy Analysis: Research and Practice* 23(1): 51–62.

[CR14] Contractor, N.S., R.C. Whitbred, F. Fonti, and C. Steglich. 2012. Understanding the ties that bind: A longitudinal investigation of the evolution of a communication network. *Western Journal of Communication* 76(4): 333–357.

[CR15] Cox, R.H., D. Dickson, and P. Marier. 2021. Resistance, innovation, and improvisation: Comparing the responses of nursing home workers to the COVID-19 pandemic in Canada and the United States. *Journal of Comparative Policy Analysis: Research and Practice* 23(1): 41–50.

[CR16] Dao, M.T., and S. Lim. 2022. Fear of disasters within the risk communication network of foreign students in Japan amid the COVID-19 pandemic crisis: A cohort design. *International Journal of Disaster Risk Reduction* 71: Article 102808. 10.1016/j.ijdrr.2022.102808 PMC8769902 35079565

[CR17] Davidovitz, M., N. Cohen, and A. Gofen. 2021. Governmental response to crises and its implications for street-level implementation: Policy ambiguity, risk, and discretion during the COVID-19 pandemic. *Journal of Comparative Policy Analysis: Research and Practice* 23(1): 120–130.

[CR18] Dunlop, C.A., E. Ongaro, and K. Baker. 2020. Researching COVID-19: A research agenda for public policy and administration scholars. *Public Policy and Administration* 35(4): 365–383.

[CR19] Evans, T. 2010. *Professional discretion in welfare services: Beyond street-level bureaucracy*. London: Ashgate.

[CR20] Gofen, A., and G. Lotta. 2021. Street-level bureaucrats at the forefront of pandemic response: A comparative perspective. *Journal of Comparative Policy Analysis: Research and Practice* 23(1): 3–15.

[CR21] Gofen, A., G. Lotta, and M.M. da Costa. 2021. Working through the fog of a pandemic: Street-level policy entrepreneurship in times of crises. *Public Administration* 99(3): 484–499. 10.1111/padm.12745 PMC8242824 34226766

[CR22] Granovetter, M. 1985. Economic action and social structure. *American Journal of Sociology* 91(3): 481–510.

[CR23] Harrits, G.S., and M.Ø. Møller. 2014. Prevention at the front line: How home nurses, pedagogues, and teachers transform public worry into decisions on special efforts. *Public Management Review* 16(4): 447–480.

[CR24] Heo, J., J.A. Park, D. Han, H.J. Kim, D. Ahn, B. Ha, W. Seog, and Y.R. Park. 2020. COVID-19 outcome prediction and monitoring solution for military hospitals in South Korea: Development and evaluation of an application. *Journal of Medical Internet Research* 22(11): Article e22131. 10.2196/22131 PMC7644266 33048824

[CR25] Hupe, P., and M. Hill. 2007. Street-level bureaucracy and public accountability. *Public Administration* 85(2): 279–299.

[CR26] Hupe, P., M. Hill, and A. Buffat, eds. 2015. In *Understanding street-level bureaucracy*. Bristol: Policy Press.

[CR27] Lee, K.-H., S. Lim, and J. Park. 2016. Expelled uninsured patients in a less-competitive hospital market in Florida, USA. *International Journal for Equity in Health* 15: Article 85. 10.1186/s12939-016-0375-z PMC4893265 27262483

[CR28] Levy, J.A., and B.A. Pescosolido, eds. 2002. In *Social networks and health*. Boston: JAI Press.

[CR75] Lim, S. 2021. *Policy network ties in the dynamic process of environmental conflict resolution: Uncovering the evolution of environmental governance*. Cham, Switzerland: Springer. 10.1007/978-3-030-70855-9

[CR29] Lim, S., and H. Nakazato. 2019. Co-evolving supportive networks and perceived community resilience across disaster-damaged areas after the Great East Japan Earthquake: Selection, influence, or both?. *Journal of Contingencies and Crisis Management* 27(2): 116–129.

[CR30] Lim, S., and H. Nakazato. 2020. The emergence of risk communication networks and the development of citizen health-related behaviors during the COVID-19 pandemic: Social selection and contagion processes. *International Journal of Environmental Research and Public Health* 17(11): Article 4148. 10.3390/ijerph17114148 PMC7312553 32532029

[CR31] Lim, S., F.S. Berry, and K.-H. Lee. 2016. Stakeholders in the same bed with different dreams: Semantic network analysis of issue interpretation in risk policy related to mad cow disease. *Journal of Public Administration Research and Theory* 26(1): 79–93.

[CR32] Lima-Silva, F., T.L. Sandim, G.M. Magri, and G. Lotta. 2020. Street-level bureaucracy in the pandemic: The perception of frontline social workers on policy implementation. *Revista de Administração Pública* 54(5): 1458–1471.

[CR33] Lipsky, M. 1980. *Street-level bureaucracy: Dilemmas of the individual in public service*. New York: Russell Sage Foundation.

[CR34] Lotta, G.S., and E.C. Marques. 2020. How social networks affect policy implementation: An analysis of street-level bureaucrats’ performance regarding a health policy. *Social Policy & Administration* 54(3): 345–360.

[CR35] Lynn, L.E., Jr., C.J. Heinrich, and C.J. Hill. 2001. *Improving governance: A new logic for empirical research*. Washington, DC: Georgetown University Press.

[CR36] Mao, X., O.W.M. Fung, X. Hu, and A.Y. Loke. 2018. Psychological impacts of disaster on rescue workers: A review of the literature. *International Journal of Disaster Risk Reduction* 27: 602–617.

[CR37] Maynard-Moody, S., and M. Musheno. 2000. State agent or citizen agent: Two narratives of discretion. *Journal of Public Administration Research and Theory* 10(2): 329–358.

[CR38] McPherson, J.M., L. Smith-Lovin, and J.M. Cook. 2001. Birds of a feather: Homophily in social networks. *Annual Review of Sociology* 27: 415–444.

[CR39] Metselaar, E.E. 1997. *Assessing the willingness to change: Construction and validation of the DINAMO*. Doctoral dissertation, Free University of Amsterdam, Amsterdam, the Netherlands.

[CR40] Meza, O., E. Pérez-Chiqués, S.A. Campos, and S.V. Castro. 2021. Against the COVID-19 pandemic: Analyzing role changes of healthcare street-level bureaucrats in Mexico. *Journal of Comparative Policy Analysis: Research and Practice* 23(1): 109–119.

[CR41] MOHS (Ministry of Health and Sports). 2019a. *Coronavirus disease 2019a situation report-10*. Naypyitaw, Myanmar: MOHS.

[CR42] MOHS (Ministry of Health and Sports). 2019b. *Coronavirus disease 2019b situation report-45*. Naypyitaw, Myanmar: MOHS.

[CR43] MOHS (Ministry of Health and Sports). 2020a. *Coronavirus disease 2020a situation report-220*. Naypyitaw, Myanmar: MOHS.

[CR44] MOHS (Ministry of Health and Sports). 2020b. *Coronavirus disease 2020b situation report-262*. Naypyitaw, Myanmar: MOHS.

[CR45] Mojica Méndez, D., S. Michelsen Gómez, and Y. Cadena Camargo. 2021. Healthcare workers during the COVID-19 pandemic from a street-level bureaucracy perspective: A narrative review of literature. *Universitas Medica* 62(4): 135–150.

[CR46] Møller, M.Ø. 2021. The dilemma between self-protection and service provision under Danish Covid-19 guidelines: A comparison of public servants’ experiences in the pandemic frontline. *Journal of Comparative Policy Analysis: Research and Practice* 23(1): 95–108.

[CR47] Molm, L.D., and K.S. Cook. 1995. Social exchange and exchange networks. In *Sociological perspectives on social psychology*, ed. K.S. Cook, G.A. Fine, and J.S. House, 209–235. Boston: Allyn & Bacon.

[CR48] Monge, P.R., and N.S. Contractor. 2003. *Theories of communication networks*. New York: Oxford University Press.

[CR49] Musheno, M., B.V. Musheno, and M. Austin. 2021. Exploring the prevalence and meaning of frontline work in the COVID-19 era: Implications for policy analysis. *Journal of Comparative Policy Analysis: Research and Practice* 23(1): 30–40.

[CR50] MWD (Myawady). 2020a. As COVID-19 prevention, control and treatment is national concern, awareness campaign must be conducted continuously and requirements must be fulfilled in time through coordination. *Myawady News*, 15 September 2020a. https://myawady.net.mm/node/1851. Accessed 28 Dec 2020a.

[CR51] MWD (Myawady). 2020b. Aids for prevention, treatment and control of COVID-19, hospital equipment donated to Coco Island Township People’s Hospital. *Myawady News*, 20 July 2020b. https://www.myawady.net.mm/node/242. Accessed 2 Jan 2021.

[CR52] Myomin, T., and S. Lim. 2022. The emergence of multiplex dynamics between information provision ties and rescue collaboration ties in disaster response settings: A longitudinal network analytic approach to flooding cases in Myanmar. *Natural Hazards*. 10.1007/s11069-022-05406-8.

[CR53] Nakazato, H., and S. Lim. 2016. Evolutionary process of social capital formation through community currency organizations: The Japanese case. *VOLUNTAS: International Journal of Voluntary and Nonprofit Organizations* 27(3): 1171–1194.

[CR54] Nakazato, H., and S. Lim. 2017. Community rebuilding processes in a disaster-damaged area through community currency: The pilot project of “Domo” in Kamaishi Japan. *Disaster Prevention and Management* 26(1): 79–93.

[CR55] OCHA (United Nations Office for the Coordination of Humanitarian Affair). 2020. Myanmar: COVID-19 situation report No. 10. https://reliefweb.int/sites/reliefweb.int/files/resources/Myanmar_COVID-19_OCHA _Situation _ Report_5Oct2020.pdf. Accessed 20 Dec 2020.

[CR56] OECD (Organization for Economic Co-operation and Development). 2020. *The territorial impact of COVID-19: Managing the crisis across levels of government*. Paris: Organisation for Economic Co-operation and Development. https://read.oecd-ilibrary.org/view/?ref=128_128287-5agkkojaaa&title=The-territorial-impact-of-covid-19-managing-the-crisis-across-levels-of-government. Accessed 27 Dec 2020.

[CR57] Paton, D. 2006. Critical incident stress risk in police officers: Managing resilience and vulnerability. *Traumatology* 12(3): 198–206.

[CR58] Pérez-Chiqués, E., P. Strach, and K. Zuber. 2021. Competing emergencies: A policy analysis of the Opioid Epidemic during COVID-19. *Journal of Comparative Policy Analysis: Research and Practice* 23(1): 85–94.

[CR59] Prottas, J.M. 1979. *People processing: The street-level bureaucrat in public service bureaucracies*. Lexington, MA: Lexington Books.

[CR60] Ripley, R.M., T.A.B. Snijders, Z. Boda, A. Vörös, and P. Preciado. 2022. *Manual for RSiena*. University of Oxford and University of Groningen.

[CR61] Seddighi, H. 2020. COVID-19 as a natural disaster: Focusing on exposure and vulnerability for response. *Disaster Medicine and Public Health Preparedness* 14(4): e42–e43. 10.1017/dmp.2020.279 PMC7492580 32713408

[CR62] Sifaki-Pistolla, D., V.E. Chatzea, S.A. Vlachaki, E. Melidoniotis, and G. Pistolla. 2017. Who is going to rescue the rescuers? Post-traumatic stress disorder among rescue workers operating in Greece during the European refugee crisis. *Social Psychiatry and Psychiatric Epidemiology* 52(1): 45–54. 10.1007/s00127-016-1302-8 27785533

[CR63] Tummers, L. 2011. Explaining the willingness of public professionals to implement new policies: A policy alienation framework. *International Review of Administrative Sciences* 77(3): 555–581.

[CR64] Tummers, L., and V. Bekkers. 2014. Policy implementation, street-level bureaucracy and the importance of discretion. *Public Management Review* 16(4): 527–547.

[CR65] Tummers, L., B. Steijn, and V. Bekkers. 2012. Explaining the willingness of public professionals to implement public policies: Content, context, and personality characteristics. *Public Administration* 90(3): 716–736.

[CR66] U.S. Embassy in Burma. 2020. COVID-19 information. https://mm.usembassy.gov/covid-19-information/. Accessed 6 Dec 2020.

[CR67] Van Roekel, H., I.M. Van der Fels, A.B. Bakker, and L.G. Tummers. 2021. Healthcare workers who work with COVID-19 patients are more physically exhausted and have more sleep problems. *Frontiers in Psychology* 11: Article 625626. 10.3389/fpsyg.2020.625626 PMC7820541 33488489

[CR68] Walker, L., and L. Gilson. 2004. We are bitter but we are satisfied: Nurses as street-level bureaucrats in South Africa. *Social Science & Medicine* 59(6): 1251–1261. 10.1016/j.socscimed.2003.12.020 15210096

[CR69] WHO (World Health Organization). 2020a. *The COVID-19 risk communication package for healthcare facilities.* Geneva: WHO. https://www.who.int/docs/default-source/coronaviruse/risk-communication-for-healthcare-facilities.pdf?sfvrsn=2a5b0e0b_2. Accessed 16 Dec 2020a.

[CR70] WHO (World Health Organization). 2020b. *COVID-19 public health emergency of international concern (PHEIC) global research and innovation forum*. Geneva: WHO. https://www.who.int/publications/m/item/covid-19-public-health-emergency-of-international-concern-(pheic)-global-research-and-innovation-forum. Accessed 30 Dec 2020b.

[CR71] Wukich, C., Q. Hu, and M.D. Siciliano. 2019. Cross-sector emergency information networks on social media: Online bridging and bonding communication patterns. *American Review of Public Administration* 49(7): 825–839.

[CR72] Yousefi Nooraie, R., J.E. Sale, A. Marin, and L.E. Ross. 2020. Social network analysis: An example of fusion between quantitative and qualitative methods. *Journal of Mixed Methods Research* 14(1): 110–124.

[CR73] Zaw, T.N., and S. Lim. 2017. The military’s role in disaster management and response during the 2015 Myanmar floods: A social network approach. *International Journal of Disaster Risk Reduction* 25: 1–21.

[CR74] Zhang, L., H. Li, and K. Chen. 2020*.* Effective risk communication for public health emergency: Reflection on the COVID-19 (2019-nCoV) outbreak in Wuhan, China. *Healthcare* 8(1): Article 64. 10.3390/healthcare8010064 PMC7151105 32245157

